# Striking differences in virulence, transmission and sporocyst growth dynamics between two schistosome populations

**DOI:** 10.1186/s13071-019-3741-z

**Published:** 2019-10-16

**Authors:** Winka Le Clecʼh, Robbie Diaz, Frédéric D. Chevalier, Marina McDew-White, Timothy J. C. Anderson

**Affiliations:** Texas Biomedical Research Institute, P.O. Box 760549, San Antonio, Texas 78245 USA

**Keywords:** *Schistosoma mansoni*, *Biomphalaria glabrata*, Life-history traits, Virulence, Transmission, Hemoglobin rate, Laccase-like activity, Survival

## Abstract

**Background:**

Parasite traits associated with transmission success, such as the number of infective stages released from the host, are expected to be optimized by natural selection. However, in the trematode parasite *Schistosoma mansoni*, a key transmission trait, i.e. the number of cercariae larvae shed from infected *Biomphalaria* spp. snails, varies significantly within and between different parasite populations and selection experiments demonstrate that this variation has a strong genetic basis. In this study, we compared the transmission strategies of two laboratory schistosome population and their consequences for their snail host.

**Methods:**

We infected inbred *Biomphalaria glabrata* snails using two *S. mansoni* parasite populations (SmBRE and SmLE), both isolated from Brazil and maintained in the laboratory for decades. We compared life history traits of these two parasite populations by quantifying sporocyst growth within infected snails (assayed using qPCR), output of cercaria larvae and impact on snail host physiological response (i.e. hemoglobin rate, laccase-like activity) and survival.

**Results:**

We identified striking differences in virulence and transmission between the two studied parasite populations. SmBRE (low shedder (LS) parasite population) sheds very low numbers of cercariae and causes minimal impact on the snail physiological response (i.e. laccase-like activity, hemoglobin rate and snail survival). In contrast, SmLE (high shedder (HS) parasite population) sheds 8-fold more cercariae (mean ± SE cercariae per shedding: 284 ± 19 *vs* 2352 ± 113), causes high snail mortality and has strong impact on snail physiology. We found that HS sporocysts grow more rapidly inside the snail host, comprising up to 60% of cells within infected snails, compared to LS sporocysts, which comprised up to 31%. Cercarial production is strongly correlated to the number of *S. mansoni* sporocyst cells present within the snail host tissue, although the proportion of sporocyst cells alone does not explain the low cercarial shedding of SmBRE.

**Conclusions:**

We demonstrated the existence of alternative transmission strategies in the *S. mansoni* parasite consistent with trade-offs between parasite transmission and host survival: a “boom-bust” strategy characterized by high virulence, high transmission and short duration infections and a “slow and steady” strategy with low virulence, low transmission but long duration of snail host infections.
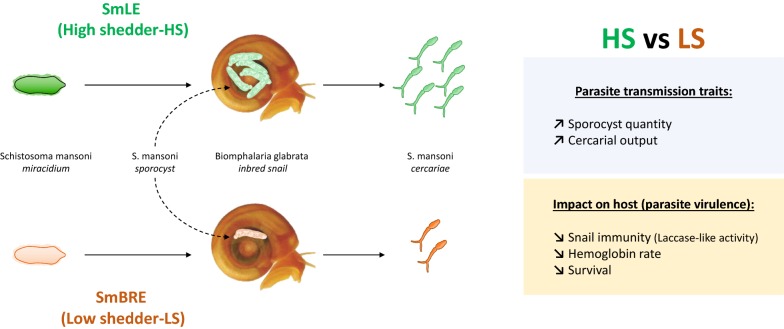

## Background

Models predicting the evolution of virulence for parasites transmitted horizontally assume generally that transmission rate (i.e. the probability for an infected host to infect a susceptible new host) and virulence (i.e. the increase in host mortality and morbidity due to infection) are positively correlated, as higher production of infective stages may be more harmful for the host [1–4]. For most of the virulence evolution models, such a trade-off shapes the relationship between parasite transmission and host survival (the higher the virulence of the parasite, the shorter the host survival and in turn the parasite lifespan) [5] for both micro- [6] and macroparasites [7]. This transmission/virulence trade-off model provides a general and intuitive framework for understanding within-species variation in parasite virulence [5, 8].

Studying production of larvae of schistosome, a human trematode parasite, offers the dual benefits of empirically testing the trade-off model for a macroparasite and improving our understanding of a key transmission-related trait in a biomedically important helminth parasite. Schistosomes infect over 200 million people in 78 countries [9], causing schistosomiasis. Among parasitic diseases, schistosomiasis ranks second behind malaria in terms of morbidity and mortality; there is no licensed vaccine and only one drug (Praziquantel) is available to treat patients. Schistosome parasites have a complex life-cycle, involving a freshwater snail (intermediate host) and a mammal (definitive host). When parasite eggs are expelled with mammal feces or urine into the water, they hatch, releasing free-living miracidia that actively search for the snail intermediate host. Larvae penetrate the snail head–foot, differentiate into mother sporocysts and then asexually proliferate to generate daughter sporocysts. This intramolluscan parasite stages feed on snail tissues such as the hepatopancreas and the albumen gland [10–12]. These organs are involved in the protein and egg production and schistosome infection results in castration of infected snails [12]. After approximately a month of infection, daughter sporocysts start to release cercariae, the mammal infective larval stage of the parasite. These exit through the snail body wall and are released into the water. This complex life-cycle can be maintained in the laboratory using rodent definitive hosts and freshwater snail intermediate hosts.

Lewis et al. [13] measured production of *S. mansoni* cercariae from infected *B. glabrata* snails in the laboratory and determined that this transmission trait varies significantly within and between different parasite populations. Moreover, Gower & Webster [14] performed replicated selection experiments in the laboratory and showed that cercarial shedding from the snail host responded extremely rapidly to selection, with a 7-fold change in cercarial production within three generations. These observations suggest that variation in transmission stage production in *S. mansoni* has a strong genetic basis. Following the transmission/virulence trade-off model, we hypothesized that *S. mansoni* parasites producing many cercariae will negatively affect snail health and cause high virulence. Virulence could occur because intramolluscan schistosome stages consume host tissue to produce large numbers of cercariae, because cercariae damage tissue when they are released from snails or because the snail energetic resources are reallocated from reproduction to defense (e.g. reactive oxygen species, ROS) in response to parasite infection may also damage host tissues [15, 16]. On the other hand, parasites that produce less larvae, but for a longer period of time, will be less virulent toward their snail host and have a lower negative impact on their physiology and survival.

In this study, we investigated the transmission and virulence of two laboratory *S. mansoni* populations both originating from South America. We observed striking differences in the number of cercariae produced by these two populations of schistosome parasites and showed that these transmission-related life-history traits have a genetic basis. We then investigated why cercarial production varies between these two populations by investigating growth of sporocysts within each infected snail. Finally, we highlight a negative relationship between transmission stage production and snail survival, health and immune parameters. Our results support the presence of a virulence/transmission trade-off in *S. mansoni*/*B. glabrata*.

## Methods

### *Biomphalaria glabrata* snails and *Schistosoma mansoni* parasites

Uninfected inbred albino *B. glabrata* snails (line Bg26 derived from 13-16-R1 line) [17] were reared in 10-gallon aquaria containing aerated freshwater at 26–28 °C on a 12L-12D photocycle and fed *ad libitum* on green-leaf lettuce. All snails used in this study had a shell diameter between 8 and 10 mm, as snail size can influence cercarial outcome [18, 19]. For all the experiments presented in this study, we used inbred snails to minimize the impact of snail host genetic background on the parasite life-history traits. *Biomphalaria glabrata* snails Bg26 were inbred over 3 generations through selfing [17], so their genomes are expected to be 87.5% identical by descent.

The SmLE schistosome population (high shedder, HS) was originally obtained by Dr. J. Pellegrino from an infected patient in Belo Horizonte (Minas Gerais, Brazil) in 1965 and has since been maintained in laboratory [13], using *B. glabrata* NMRI population as intermediate host and Syrian golden hamster (*Mesocricetus auratus*) as definitive hosts. The SmBRE schistosome population (low shedder, LS) was sampled in the field in 1975 from Recife (East Brazil) [20] and has been maintained in the laboratory in its sympatric albino Brazilian snail host BgBRE using hamsters or mice as the definitive host.

### Measurement of *S. mansoni* life-history traits and virulence

**Fig. 1:**
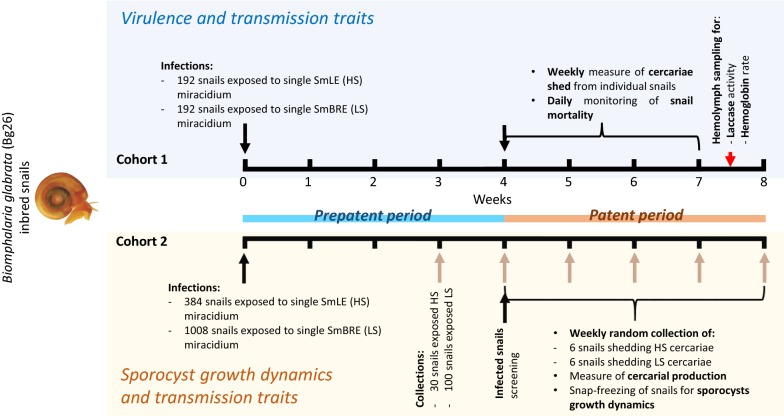
Outline of the experimental design. We used two independent cohorts of *Biomphalaria glabrata* Bg26 inbred snails. Each snail was exposed to one miracidium from the SmLE (HS) or SmBRE (LS) *Schistosoma mansoni* populations. In Cohort 1, we measured transmission stage production for SmLE (HS) and SmBRE (LS) populations during 4 weeks of the patent period (week 4 to 7 post-infection). We also evaluated the virulence of these two populations of parasite by measuring the daily snail survival during the patent period. After 7.5 weeks post-infection, surviving infected snails were bled and we measured the total laccase-like activity as well as the hemoglobin rate in the collected hemolymph samples. We used Cohort 2 to determine the weekly sporocyst growth dynamics in snails for the late prepatent (week 3) and the patent period (week 4 to 8)

We compared larval output (i.e. cercarial production) and intramolluscan development (i.e. sporocyst development and growth) of SmLE (HS) and SmBRE (LS) parasite populations using the same inbred snail population in two independent cohort experiments (Fig. 1). We also measured the impact of these parasitic infections on the snail host by quantifying snail survival and physiological responses (i.e. laccase-like activity and hemoglobin rate in the hemolymph).

#### Cohort 1: *S. mansoni* cercarial output and impact on snail survival and physiological response

(i) ***S. mansoni*** **cercarial production over time**. To compare cercarial production over the time for the SmLE (HS) and SmBRE (LS) parasite populations, we exposed each of 384 inbred *B. glabrata* Bg26 to a single miracidium. Miracidia of each population were hatched from eggs recovered from 45-day-infected female hamster livers infected. The livers were homogenized and the eggs were filtered, washed with normal saline [154 mM Sodium chloride (Sigma-Aldrich, St-Louis, MO, USA), pH 7.5], transferred to a beaker containing freshwater and exposed to artificial light (1 h) to induce hatching. We exposed individual snails (192 per parasite populations) to single miracidium in 24-well plates for at least 4 h and exposed snails were maintained in trays (48 per tray) for 4 weeks. We used single miracidium for infections to avoid competition effects and obtain the phenotype corresponding to a single parasite genotype. We covered trays with black plexiglass lids after 3 weeks to reduce cercarial shedding. At 4 weeks post-exposure, we placed snails in 1 ml of freshwater in 24-well plates under artificial light for 2 h to induce cercarial shedding. Shedding was conducted late morning to early afternoon. We isolated each infected snail in a 100 ml glass-beaker filled with ~ 50 ml of freshwater and kept them in the dark until week 7 post-infection. We replaced freshwater as needed (typically every 2 days) and fed snails *ad libitum*. To quantify cercarial shedding, we placed infected snails in 1 ml of freshwater in a 24-well plate under artificial light (as described above) every week for 4 weeks (week 4 to 7). For each well, we sampled three 100 µl aliquots, added 20 µl of 20× normal saline and counted the immobilized cercariae under a microscope. We multiplied the mean of the triplicated measurement by the dilution factor (10) to determine the number of cercariae produced by each infected snail. We also extracted DNA from week 4 cercariae to determine parasite sex by PCR [21].
(ii) **Snail survival.** We evaluated survival of the infected snails in cohort 1 over the course of the infection. We compared the survival of infected snails with a group of 48 uninfected control snails. The uninfected control snails were sham-infected by placing them in 24-well plates but without miracidia and then treated exactly the same way as the infected snails. We monitored snail survival every day from the first cercarial shedding day (28 days after exposure) to 22 days after the first cercarial shedding (50 days after exposure).
(iii) **Snail physiological response to parasitic infection: hemolymph laccase-like activity and hemoglobin rate**. In week 7, three days after the last cercarial shedding, we collected hemolymph as described by Le Clec’h et al. [22] from all surviving snails infected with SmLE (HS) and SmBRE (LS), as well as from uninfected Bg26 controls maintained under the same conditions. We measured both laccase-like activity and the hemoglobin (protein carrying oxygen in *B. glabrata* hemolymph) rates in the hemolymph of each snail infected with SmLE (HS), SmBRE (LS) or uninfected control. While phenoloxidase (PO) activity is known to be involved in invertebrate immunity, the specific role of laccase activity in snails is currently unknown [22]. However, laccase is overexpressed in infected snails [23] suggesting a potential role in immunity.

We measured the total laccase-like activity as described in Le Clec’h et al. [22]. In brief, we combined 10 µl of freshly collected hemolymph to 40 µl of cacodylate buffer and 40 µl of bovine trypsin (1 mg/ml) in a 96-well optical plate (Corning, Corning, NY, USA). Each sample was coupled to a control, where 10 µl of the same hemolymph aliquot was combined to 40 µl of 10 mM diethylthiocarbamate (a specific inhibitor of PO enzymes) and 40 µl of bovine trypsin. We incubated plates in the dark, at 37 °C for 45 min, then added 120 µl of the freshly prepared *p*-phenylenediamine substrate (50 mM). After 2 h incubation at 37 °C, before reaching the plateau-phase of the laccase-like activity, we measured dopachrome formation at λ = 465 nm using a SpectraMax M1 (Molecular Devices, San Jose, CA, USA). A substrate auto-oxidation control was also performed for each experiment, where the hemolymph sample was replaced by 10 µl of distilled water. The value of this substrate auto-oxidation control was subtracted from sample and control values.

To quantify the hemoglobin rate, we centrifuged the hemolymph (5 min, at 300× *g* at 4 °C) to pellet the hemocytes (i.e. the immune cells), as the hemoglobin is not sequestrated in cells but free in the plasma [24]. We collected the plasma in a fresh microtube placed on ice. The hemoglobin rate was determined by measuring the optical density (OD) of the hemoglobin solution at λ = 410 nm, the maximum absorption of *B. glabrata* hemoglobin [24]. In a 96-well optical plate (Corning), we combined 190 µl of PBS 1× to 10 µl of plasma. A blank control, containing only 200 µl of PBS 1× was also performed for each assay. The value obtained for this blank control was subtracted from the sample wells (i.e. containing plasma).

#### Cohort 2: *S. mansoni* sporocyst time course and growth dynamics

(i) **Cohort design.** Evaluating sporocyst development within infected snails requires the sacrifice of snails at multiple time points. We exposed a second cohort of 1392 Bg26 inbred snails to single miracidium from our two *S. mansoni* populations. We exposed 384 snails to SmLE (HS) and 1008 to SmBRE (LS). The numbers are unequal because SmLE (HS) shows much higher infection rates than SmBRE (LS). Infection rates assessed at week 4 post-exposure (after the first cercarial shedding) were 76/207 surviving snails for SmLE (HS) compared to 36/550 surviving snails for SmBRE (LS). Snails were kept in trays (48 per tray). We sampled snails at week 3 post-exposure, one week prior to cercarial maturation. To ensure sampling of infected snails, we randomly picked 30 snails exposed to SmLE (HS) and 100 snails exposed to SmBRE (LS).
  In week 4, we placed each infected snail in 1 ml of freshwater in 24-well plates under artificial light for 2 h to induce cercarial shedding and identify infected snails. From week 4 to week 8, we isolated infected snails in trays (48 per tray) under a black lid. Each week, we randomly picked 6 infected snails from each parasite population and counted the cercariae released by each snail as described above. We cleaned the shell of sampled snails with 70% ethanol, snap-froze them individually in liquid nitrogen and store them at − 80 °C for further molecular analysis.
(ii) **gDNA extractions from exposed snails.** To prepare exposed snails for molecular analysis, we crushed snails individually in a sterile, liquid nitrogen-cooled mortar and pestle to create a fine, homogenized tissue powder and kept 100 µl of this powder into 1.5 ml tubes at − 80 °C until gDNA extraction. We extracted the gDNA using a DNeasy Blood & Tissue Kit (Qiagen, Germantown, MD, USA) according to manufacturer instructions, with tissue lysis for 20 min at 56 °C. We quantified the gDNA using a Qubit dsDNA BS Assay Kit (Invitrogen, Carlsbad, CA, USA).
(iii) **Multiplex PCR to identify infected snails.** To screen for infected prepatent snails (week 3, Fig. 1), we performed a multiplex PCR on the gDNA recovered from snails’ powder. We used the *α-tubulin 2 S. mansoni* gene (GenBank: S79195.1; gene number Smp_103140) [25] as specific parasite marker and the P-element induced wimpy testis (*piwi*) gene from *B. glabrata* [26] as specific snail host marker. We identified the *piwi* gene (BGLTMP009852) in the *B. glabrata* genome (BglaB1 assembly) using the blast module of VectorBase [27] and 3 ESTs showing similarities with *piwi* (GenBank: FC855819, FC856421 and FC856380).
  Multiplex PCR reactions consisted of 8.325 µl sterile water, 1.5 µl 10× buffer, 1.2 µl dNTP (2.5 mM each), 0.9 µl MgCl_2_, 0.5 µl of each primer (10 µM) for both markers (*piwi* F: 5′-CTT CTC CAA TGC TAC CAT CAA AG-3′; *piwi* R: 5′-TTT CAT CCT CCA CAC TGA CAA-3′; *α-tubulin 2* F: 5′-CGA CTT AGA ACC AAA TGT TGT AGA-3′; *α-tubulin 2* R: 5′-GTC CAC TAC ATT GAT CCG CT-3′), 0.075 µl of *Taq* polymerase (TaKaRa, Mountain view, CA, USA) and 1 µl of gDNA template using the following program: 95 °C for 5 min, (95 °C for 30 s, 55 °C for 30 s and 72 °C for 30 s) × 35 cycles, 72 °C for 10 min. Infected snails exhibit a two-band pattern at 361 bp and 190 bp on an agarose gel, while uninfected snails show one band at 361 bp (Additional file 1: Figure S1). All the primers were designed using PerlPrimer v1.21.1 [28].
(iv) **qPCR to quantify the proportion of sporocyst cells within infected snails.** The daughter sporocysts that release cercariae are intertwined in the snail tissue, making them difficult to isolate and study, so they have been neglected relative to other parasite life-stages. Using a custom quantitative PCR assay, we quantified the relative proportion of parasite cells within infected snail at different time-points of the infection. This qPCR assay provides a relative measure of parasite growth within infected snails.

We quantified a single copy gene from the parasite (*α-tubulin 2*, see above [25]) and from the snail (*piwi*, see above [26]). The qPCR assay used a different set of *piwi* primers (*piwi* F: 5′-AAT CAT CTC ATT CAA CCT GTC CAT-3′ and *piwi* R: 5′-ATT TCC GCC ATC ATA GCC C-3′) amplifying a 107-bp amplicon and the same *α-tubulin 2* primers as described in the end-point PCR assay. The qPCR primers amplify a smaller *piwi* amplicon than those used for the end-point PCR to ensure comparable efficiency to the *α-tubulin 2* amplicon. We conducted qPCR in duplicate for each reaction (i.e. samples and standards). Reactions consisted of 5 µl SYBR Green PCR master mix (Applied Biosystems, Foster City, CA, USA), 3.4 µl sterile water, 0.3 µl of each primer (10 µM) and 1 µl of standard PCR product or sample gDNA. We used the following program: 95 °C for 10 min (95 °C for 15 s and 60 °C for 1 min) × 40 cycles followed by a melting curve step (15 s at 95 °C and then rising in 0.075 °C increments/s from 60 °C to 95 °C), to check for the uniqueness of the product amplified. We plotted standard curves using seven 10-fold dilutions of a purified *α-tubulin 2* PCR product for *S. mansoni* parasite (*α-tubulin 2* copies/µl: 2.69 × 10^1^ − 2.69 × 10^7^) and seven 10-fold dilutions of a purified *piwi* PCR product for *B. glabrata* (*piwi* copies/µl: 2.60 × 10^1^ − 2.60 × 10^7^). PCR products for standard curves were generated using TaKaRa Taq R001 AM Kit (Clonetech- TaKaRa, Mountain view, CA, USA) and the manufacturer’s protocol [PCR cycles: 95 °C for 5 min, (95 °C for 30 s, 60 °C for 30 s, 72 °C for 30 s) × 35 cycles, 72 °C for 10 min], purified using SigmaSpin Sequencing Reaction Clean-Up Kit (Sigma-Aldrich) following the manufacturer’s protocol and quantified using Qubit dsDNA BR Assay Kit (Invitrogen). We estimated the number of copies in the PCR products as follows: PCR product length × [average molecular mass of nucleotides (330 g/mol) × 2 strands] × Avogadro constant. The number of *α-tubulin 2* and *piwi* copies in each sample was estimated according to the standard curve (QuantStudio Design and Analysis Software, Applied Biosystems, Foster City, CA, USA). Both snail and parasite genes quantified are present as a single-copy gene, so the number of gene copies quantified corresponds to the number of genomes of each organism. As both parasite and snail are diploid, the number of genomes is directly proportional to the number of cells from each organism. The proportion of parasite cells within infected snails, our relative measure of parasite growth, was calculated as follows:

$$Proportion_{parasite} = {\text{ N}}_{\text{parasite}} /\left( {{\text{N}}_{\text{parasite}} + {\text{N}}_{\text{snail}} } \right)$$

where N is the number of parasite or snail cells measured by qPCR.

### Statistical analysis

All statistical analyzes and graphs were performed using R software (version 3.5.1). When data were not normally distributed (Shapiro test, *P* < 0.05), results were compared with a Kruskal–Wallis followed by Dunn’s multiple comparison test or simple pairwise comparison (Mann–Whitney U-test for unpaired data or Wilcoxon test for paired data). When data followed a normal distribution, results were compared with a simple pairwise comparison Welsh t-test. We used binomial tests to analyze sex ratio differences. We performed survival analysis using log-rank tests (R survival package) and correlations analysis with Pearson’s test. The confidence interval of significance was set to 95% and *P*-values < 0.05 were considered as significant.

## Results

### Striking differences in transmission stage production between two *Schistosoma mansoni* populations

**Fig. 2:**
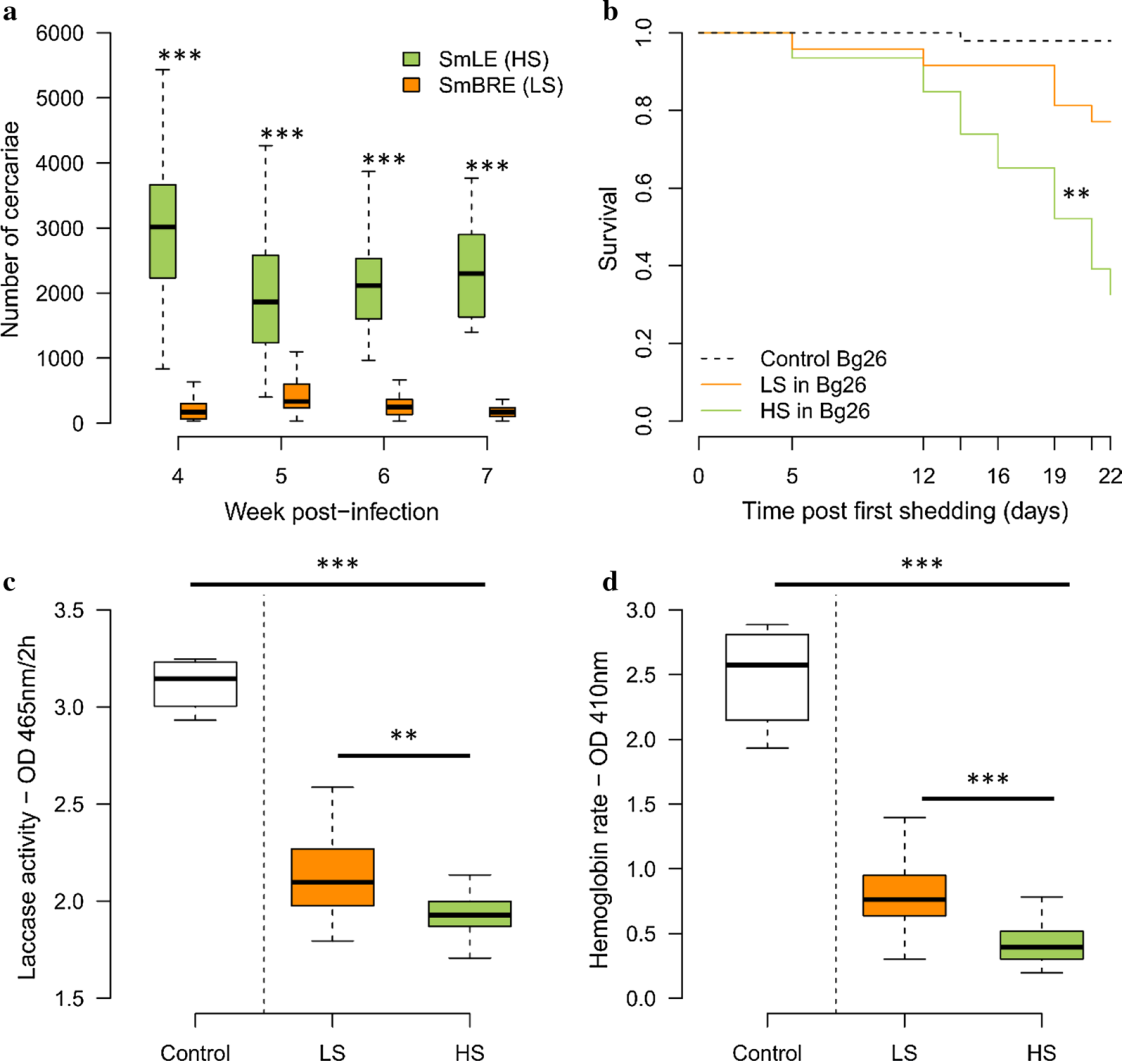
Transmission stage production and virulence of two *Schistosoma mansoni* populations [SmLE (HS) and SmBRE (LS)]. **a** Difference in the number of cercariae produced by SmLE (HS) and SmBRE (LS) *S. mansoni* populations during 4 weeks of the patent period (week 4 to 7 post infection). SmLE (HS) population was shedding more cercariae than SmBRE (LS) population of parasite at all the time points. **b** Survival of the infected and control *Biomphalaria glabrata* (Bg26 inbred) snails from the first day of cercarial shedding to day 22 after the first shedding. Infection with SmLE (HS) parasites resulted in greater snail mortality than infection with SmBRE (LS) parasites. **c** Infected snails showed a decrease in laccase-like activity in the snail hemolymph compared to uninfected ones. Snails infected with SmLE (HS) parasites showed a greater decrease than that in snails parasitized by SmBRE (LS) parasites. **d** The overall hemoglobin rate in the hemolymph was reduced by the presence of schistosome parasites. However, the reduction was greater when snails are infected with the SmLE (HS) parasites. *P < 0.05, **P ≤ 0.01, ***P ≤ 0.001

**Fig. 3:**
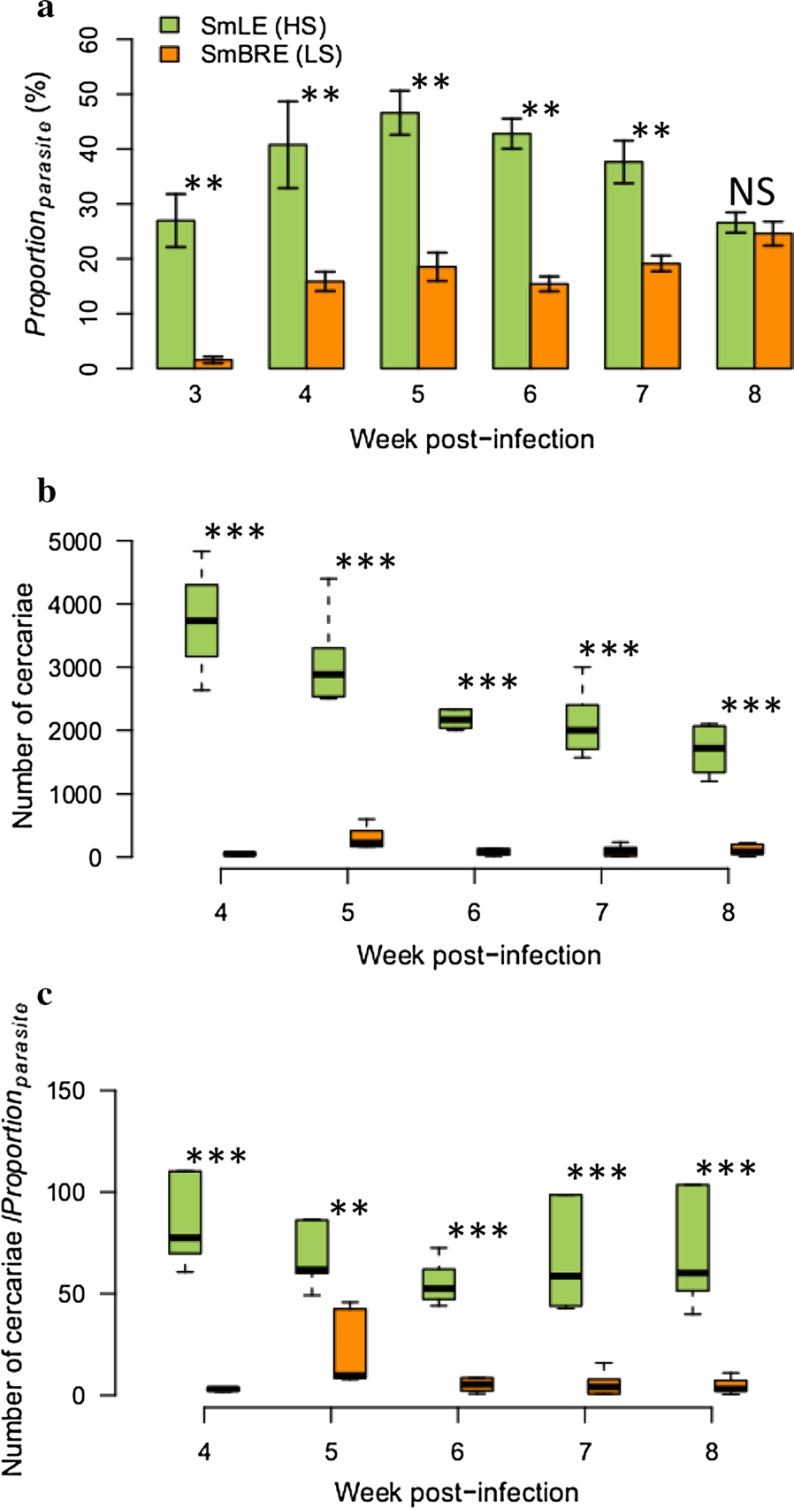
Sporocyst growth dynamics and cercarial production in SmLE (HS) and SmBRE (LS) *S. mansoni*. **a** Comparison of the daughter sporocyst developmental kinetics for SmLE (HS) and SmBRE (LS). The proportion of sporocyst cells within snails (*Proportion*_*parasite*_) were quantified by qPCR during 6 weeks of the infection (from week 3 to 8). SmLE (HS) sporocysts grow faster and are more numerous than the SmBRE (LS) ones. **b** Cercarial shedding profiles of the SmLE (HS) and SmBRE (LS) during the 5 weeks of the patent period (i.e. cercarial shedding time) (weeks 4 to 8). SmLE (HS) parasites produced significantly more cercariae than the SmBRE (LS) parasites. **c** Ratio calculated by dividing the number of cercariae produced by the proportion of daughter sporocyst cells present in the snail, infected with SmLE (HS) or the SmBRE (LS) population. Differences in proportions of sporocyst within infected snails were not sufficient to explain the difference in cercarial output between the SmLE (HS) and SmBRE (LS) infected snails. *P < 0.05, **P ≤ 0.01, ***P ≤ 0.001

The SmLE (HS) parasite population produce more cercariae than the SmBRE (LS) population in both Cohort 1 (4 weeks of shedding) and Cohort 2 (5 weeks of shedding): on average, our SmLE (HS) population shed 8-fold more cercariae than SmBRE (LS) [Kruskal–Wallis test: Cohort 1: *χ*^2^ = 224.78, *df* = 1, *P* < 2.2 × 10^−16^ (Fig. 2a); Cohort 2: *χ*^2^ = 44.296, *df* = 1, *P* = 2.824 × 10^−11^ (Fig. 3b)]. In this experiment, all the infected snails were from the same inbred *B. glabrata* population (Bg26) to minimize the impact of the host genetic background, because we know that cercarial shedding can be influenced by the snail genotype [29].

We exposed the snail hosts to only one miracidium [SmLE (HS) or SmBRE (LS)], male or female. We were therefore able to sex each parasite that developed inside the host and test the influence of the parasite sex on the cercarial output. We found no sex-ratio bias [sex-ratio = (number of males/number of females) for SmBRE (LS) = 1.09, binomial test: *P* = 0.883; sex-ratio for SmLE (HS) = 0.84, binomial test: *P* = 0.658]. Parasite sex does not impact the cercarial production in the SmLE (HS) population (Mann–Whitney U-test, *U*_(44)_ = 238, *Z* = 0.529, *P* = 0.6; Additional file 2: Figure S2) but does have an influence on the SmBRE (LS) population where male LS genotypes produced significantly less cercariae than female ones (Mann-Whitney U-test, *U*_(44)_ = 154.5, *Z* = − 2.397, *P* = 0.016; Additional file 2: Figure S2).

### Increased virulence of the high shedder *S. mansoni* population

#### Comparison of survival rates in infected snails

SmLE (HS) parasites have a strongest impact on the survival of the host after the first shedding day (log-rank test global analysis: *χ*^2^ = 14.3, *df* = 2, *P* = 8 × 10^−4^) compared to SmBRE (LS) (log-rank test: *χ*^2^ = 6.4, *df* = 1, *P* = 0.011) or the control group (i.e. uninfected snails; log-rank test: *χ*^2^ = 8.1, *df* = 1, *P* = 0.004). Snails infected with SmBRE (LS) parasite did not have significantly greater mortality than controls (Fig. 2b).

#### Comparison of hemoglobin and laccase-like activity in infected snails

We also measured the impact of our two population of parasites on their snail hosts by measuring laccase-like activity in the snail hemolymph, 7.5 weeks after parasite exposure [22]. Unlike the survival data, where only SmLE (HS) population has a negative impact on the snail host, we observed that the laccase-like activity is reduced in snails infected with both SmBRE (LS) and SmLE (HS) relative to controls (Kruskal-Wallis test followed by Dunn *post-hoc* test, *χ*^2^ = 22.82, *df* = 2, *P* = 1.108 × 10^−5^; see Fig. 2c). However, snails infected with SmLE (HS) population showed greater reduction in laccase-like activity than those infected with SmBRE (LS) (Welsh t-test, *t* = 3.314, *df* = 48.25, *P* = 0.001; Fig. 2c).

We observed a similar impact of infection on hemoglobin rate, measured in hemolymph samples collected 7.5 weeks after parasite-exposure. Both SmLE (HS) and SmBRE (LS) infected snails had reduced hemoglobin relative to controls (Kruskal–Wallis test followed by Dunn *post-hoc* test, *χ*^2^ = 30.70, *df* = 2, *P* = 2.155 × 10^−7^; see Fig. 2d), while SmLE (HS) infected snails had significantly reduced hemoglobin relative to SmBRE (LS) infected snails (Mann-Whitney U-test, *U*_(50)_ = 65, *Z* = 4.281, *P* < 0.0001; Fig. 2d).

Moreover, we found a strong positive correlation between hemoglobin rate and laccase-like activity (Pearson’s *r* = 0.78, *P* = 4.633 × 10^−12^, Additional file 3: Figure S3c). These two proteins provide a good proxy of snail health and are both severely impacted by *S. mansoni*, with the impact dependent on parasite population [SmBRE (LS) or SmLE (HS)]. We observed a strong negative correlation between the hemoglobin rate and the average number of cercariae produced (Pearson’s *r* = − 0.54, *P* = 2.528 × 10^−5^, Additional file 3: Figure S3b) as well as between the laccase-like activity in the hemolymph and the average number of cercariae produced (Pearson’s *r* = − 0.33, *P* = 0.017, Additional file 3: Figure S3a). These physiological proxies of snail health support the mortality data, showing that our SmLE (HS) population of *S. mansoni* is more virulent toward the snail inbred host (Bg26) than the SmBRE (LS) population of parasites.

### Dynamics of sporocyst growth in infected snails

Cercariae are free-living schistosome larvae produced by daughter sporocysts. This parasite stage is intertwined with the snail host hepatopancreas and ovotestis, so is difficult to quantify even in dissected snails. In Cohort 2, we investigated the relationship between the quantity of cercariae released and the total quantity of sporocyst tissue developing in the snail for both SmLE (HS) and SmBRE (LS) parasites. We measured the proportion of parasite cells relative to snail cells (*Proportion*_*parasite*_) within infected snails using a custom qPCR assay. SmLE (HS) parasites have a significantly higher growth (average *Proportion*_*parasite*_ ranges from average values of 26–47% with a maximum of 60.46% for individual snails) than SmBRE (LS) parasites (average *Proportion*_*parasite*_ ranges from average values of 1.5–25%, with a maximum of 31.12% for individual snails) across the time-course experiment (Kruskal–Wallis test, *χ*^2^ = 27.417, *df* = 1, *P* = 1.64 × 10^−7^, Fig. 3a). However, at the end of the time course (week 8 post-infection), both SmBRE (LS) and SmLE (HS) sporocysts comprised approximately 25% of snail tissue (Welsh t-test, *t* = − 0.686, *df* = 8.97, *P* = 0.510; Fig. 3a). The sporocyst growth profiles also differed in shape; SmLE (HS) reached a peak in week 5 during the second week of shedding and then declined, while SmBRE (LS) was still increasing in week 7 at the end of the time-course.

Differences in *Proportion*_*parasite*_ explain some, but not all of the variation in cercarial shedding between the SmLE (HS) and SmBRE (LS) populations. At week 8, values of *Proportion*_*parasite*_ are similar for SmLE (HS) and SmBRE (LS), but SmLE (HS) still continued to produce around 15-fold more cercariae than SmBRE (LS) [mean ± SE cercariae: SmLE (HS): 1689 ± 164 *vs* SmBRE (LS): 109 ± 35]. Furthermore, in weeks 4 to 8, the differences in *Proportion*_*parasite*_ were not sufficient to explain the differences in cercarial output between the SmLE (HS) and SmBRE (LS) infected snails (Kruskal–Wallis test, *χ*^2^ = 43.09, *df* = 1, *P* = 5.22 × 10^−11^, Fig. 3c).

**Fig. 4:**
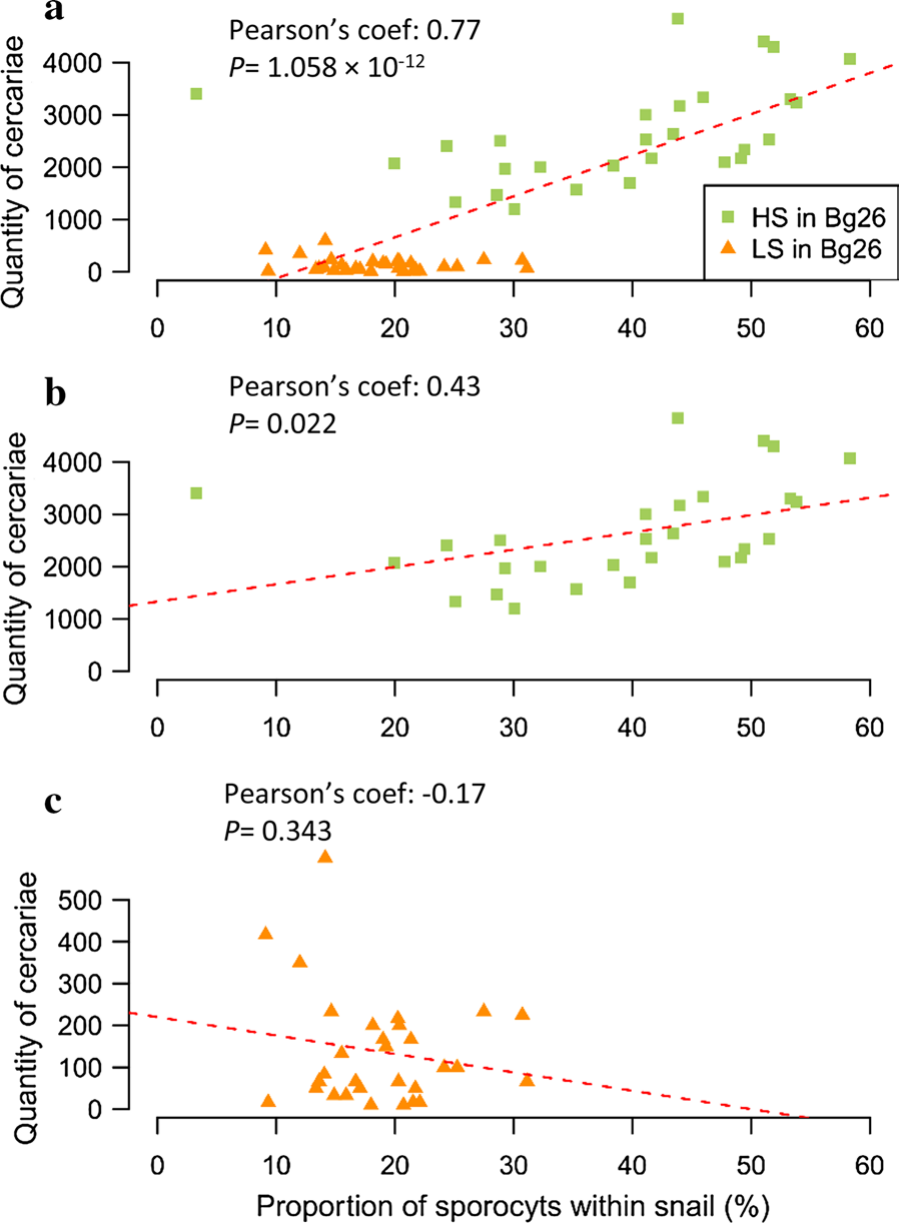
Correlations between sporocyst quantity and cercarial output for SmLE (HS) and SmBRE (LS) parasites. **a** There was a strong correlation between the proportion of sporocysts present in the snail tissue and the quantity of cercariae released by the same snail (Pearson’s *r* = 0.77, *P* = 1.058 × 10^−12^). This correlation was mainly driven by the SmLE (HS) parasite population (Pearson’s *r* = 0.43, *P* = 0.022) (**b**). **c** There was no significant correlation for the SmBRE (LS) parasite population (Pearson’s *r* = − 0.17, *P* = 0.343)

We observed a strong correlation between *Proportion*_*parasite*_ and the quantity of cercariae released by the same snail (Pearson’s *r* = 0.77, *P* = 1.058 × 10^−12^, Fig. 4c). This correlation is driven by SmLE (HS) parasite population (Fig. 4b) and there was no correlation observed for SmBRE (LS), for which there is more limited variation in the number of cercariae produced (Fig. 4c).

## Discussion

### Virulence-transmission trade-offs in *S. mansoni*

Our results are consistent with a transmission/virulence trade-off model [30–34]. We show that the quantity of sporocysts, the intramolluscan stage of *S. mansoni* and the number of cercariae shed are strongly correlated. The SmLE (HS) parasite population has a higher sporocyst growth rate within infected snails, produces large numbers of cercariae (i.e. transmission stage) and it is highly virulent toward the snail. Virulence was evident both from the high rate of snail mortality and from reduced levels of two physiological parameters: hemoglobin rate [35, 36] and laccase-like activity [22]. In contrast, the SmBRE (LS) population of *S. mansoni* has a much lower sporocyst growth rate inside the snail host, releases fewer cercariae compared to the SmLE (HS) parasite and is much less virulent for its snail host. Similar patterns of life-history variation are also observed in the water flea-bacteria (*Daphnia magna*-*Pasteuria ramosa*) infection model, a comparable system where bacterial infection castrates water fleas. In this system, water fleas infected with high virulent “early killer” spores had a significantly higher death rate compared to those infected with low virulence “late killers”. Variation in time of death was at least in part caused by genetic differences among parasites [6].

Our findings provide an interesting contrast with patterns observed in experimental work on the SmPR1 parasite population originally isolated from Puerto Rico [14, 37], where parasites with high cercarial shedding show low virulence to the snail host. This inverse relationship between cercarial shedding and virulence was initially observed by Davies et al. [37], who isolated five inbred parasites lines from the SmPR1 laboratory line. Consistent with this, experimental selection of SmPR1 populations for high or low cercarial output resulted in rapid divergence in cercarial production, with high shedding parasites showing reduced virulence to the snail host [14]. These results run counter to classical theoretical work suggesting that virulence is expected to be a by-product of increased transmission stage production [5, 30]. However, these authors also showed that high virulence to the snail host was associated with lower virulence to the mammal host. They suggested an alternative trade-off model involving pleiotropy between genes underlying parasite traits conferring fitness within the definitive (mammal) and intermediate (snail) host [14]. They suggested that such pleiotropy might explain patterns of virulence observed and promote the maintenance of genetic and phenotypic polymorphisms in parasite populations utilizing multiple hosts. The intriguing differences in transmission stage production and virulence toward the snail host observed in work with SmPR1 [14, 37] and our work, suggest that the underlying causes of high virulence in different schistosome populations may vary [38].

We also demonstrated that the sex of the parasite can impact cercarial shedding. Indeed, for the SmBRE (LS) population only, we highlighted that male genotypes of *S. mansoni* produced significantly less cercariae than the female ones. This result is consistent with observations collected by Boissier et al. [39] in their meta-analysis of Brazilian schistosome/snail systems. Whether higher production of cercariae by female SmBRE (LS) sporocysts results in higher levels of snail virulence is unclear. We observed minimal impacts of SmBRE (LS) infections on survival of infected *versus* uninfected snails. We therefore expect minimal impact of schistosome sex on virulence to snails.

### What causes virulence to snails?

We compared virulence of our two schistosome populations by directly measuring mortality of inbred snails and by quantifying laccase-like activity and hemoglobin rate. Schistosome parasites have a direct impact on their snail intermediate hosts; as they grow and generate cercariae, sporocysts deplete galactogen in the albumen gland and consume the ovotestis and hepatopancreas, converting stored glycogen to glucose [11, 12]. Wang et al. [40] found that neuropeptides and precursor proteins involved in snail reproduction were heavily downregulated in infected prepatent snails compared to uninfected snails, suggesting that this could play a role in castration of *Biomphalaria* snails by schistosomes. Other downregulated neuropeptides in prepatent snails were linked to snail feeding and growth, process that directly impact the reproductive capacity, metabolism and immunity of the snail host. The high level of mortality observed in snails infected with SmLE (HS) is consistent with the fact that sporocysts cells comprised on average 26–47% of the cells within infected snails (Cohort 2), largely replacing the hepatopancreas and ovotestis. This can also explain the reduction in laccase-like activity and hemoglobin rate in SmLE (HS) relative to SmBRE (LS) and the strong correlations between cercarial production, laccase-like activity and hemoglobin rate observed in SmLE (HS), as this rapidly growing parasite depletes snail host resources. Cercarial shedding is also harmful to snails because cercariae puncture the tegument to exit the snail, causing hemolymph loss, another potential cause of early death in infected snails (W. Le Clec’h, F. Chevalier, personal observations). This may also contribute to the higher virulence of SmLE (HS) compared with SmBRE (LS) population of parasites.

### Natural variation or an artifact of laboratory maintenance?

The two populations of parasites [SmLE (HS) and SmBRE (LS)] used in our experiments have been maintained in laboratory conditions for 54 and 44 years, respectively. We were concerned that long-term maintenance of these parasites in the laboratory could have selected for the life-history traits observed; for example, serial passage of microbial pathogens often imposes selection for rapid growth and high virulence [41–43]. Interestingly, cercarial production in SmLE and SmBRE has remained stable over multiple decades. Our SmLE (HS) population was reported to show high cercarial production compared to the other populations of *S. mansoni* parasites in a study published 33 years ago [13]. Similarly, the low shedding profile exhibited by our SmBRE (LS) population is consistent with low shedding by this *S. mansoni* population reported 26 years ago [19]. Hence, cercarial shedding phenotypes observed in these two parasite populations have remained stable over time.

Field-collected snails infected with *S. mansoni* show extensive variation in cercarial production. However, interpretation of this cercarial variation is difficult, because variation can result from the number of infecting miracidia, from environmental factors (snail nutrition etc.) and/or genetics. Further work to characterize parasite genetic variation for cercarial production in natural schistosome populations would be of great interest.

### Dynamics of sporocyst growth

We observed dramatic differences in sporocyst growth profiles between SmLE (HS) and SmBRE (LS) using our qPCR assay (Fig. 3a). These differences reflect sporocyst growth rate, rather than differences in number of infecting miracidia, because we exposed each snail to a single miracidium. The growth kinetics differs significantly: SmLE (HS) sporocysts have parabolic growth profile and a much higher proportion of daughter sporocysts produced, even during the prepatent period (i.e. 3 weeks after parasite exposure and 1 week before the first cercarial shedding). The SmLE (HS) *S. mansoni* population grows faster (reaching an average of 47% of cells within infected snails) compared to SmBRE (LS) population (reaching an average of 25% of cells within infected snails). Interestingly, the SmBRE (LS) daughter sporocyst kinetics in whole snails measured by qPCR is similar to that obtained by microscopy and 3D reconstructions of sporocysts (in the hepatopancreas only) in the same parasite population [19], with an initial exponential growth of the parasite tissues followed by a plateau.

Differences in sporocyst proportions in SmLE (HS) and SmBRE (LS) infected snails do not fully explain the difference in cercarial production between these lines. The SmBRE (LS) infected snails shed significantly fewer cercariae than predicted from qPCR measures of sporocysts cells in infected snails (Fig. 3c). We suspect that SmLE (HS) and SmBRE (LS) sporocysts may exhibit different cellular trajectories, with differences in development of cell populations that differentiate to generate cercariae and those that give birth to the next generation of daughter sporocysts [44]. Advances in our understanding of stem-cell differentiation of *S. mansoni* within the molluscan host now provide the tools needed to investigate these transmission-related developmental differences at the cellular and molecular levels [44–46].

## Conclusions

In this study, we describe two different transmission strategies of *S. mansoni* that have a strong genetic basis: a “boom-bust” strategy characterized by high virulence, high transmission and short duration infections for SmLE (HS), compared with “slow and steady” strategy with low virulence, low transmission but long duration of infection for SmBRE (LS) populations. Our empirical characterization of life-history variation in schistosomes provides valuable parameter estimates for theoretical work on ecology and evolution of parasite life-history traits [47, 48]. We speculate that these two different strategies may be selected in the field to optimize parasite transmission and fitness in different environments. We envisage that in high transmission areas, where individual snails may contain competing *S. mansoni* infections (or co-infections with other trematodes species [49], the SmLE (HS) strategy may be strongly selected as a consequence of intense within-host competition. Conversely, in low prevalence sites, where coinfections are rare, the SmBRE (LS) strategy and limited virulence to the snail host may be advantageous. Genetic crosses between parasites from these two distinctive *S. mansoni* populations, followed by a classical quantitative trait locus analysis [50], now provide the opportunity to determine the genetic basis of these key transmission-related phenotypes in an important human helminth infection.

## Supplementary information

**Additional file 1: Figure S1.** Multiplex PCR assay for identifying infected prepatent snails. We electrophoresed multiplexed PCR products generated using *piwi* and *α-tubulin-2* primers on 2% agarose gel. The size ladder used is the 100-bp ladder from Promega. Infected *B. glabrata* Bg26 snails show a “double-band”: a 361-bp *piwi* snail-specific band and a 190-bp *α-tubulin-2* parasite-specific band. Uninfected snails exhibit only the 361 bp *piwi* snail-specific band, while *S. mansoni* control show only the 190 bp *α-tubulin-2* parasite-specific band.

**Additional file 2: Figure S2.** Impact of *S. mansoni* sex on the cercarial production. Male sporocysts produced significantly less cercariae than female sporocysts in SmBRE (LS) parasite. There was no difference driven by the sex of the parasites for the SmLE (HS) population of *S. mansoni*. *P < 0.05, **P ≤ 0.01, ***P ≤ 0.001.

**Additional file 3: Figure S3.** Virulence of* S. mansoni* parasites; correlation between cercarial production and measured* B. glabrata* snail physiological parameters. a There was a negative correlation between the average number of cercariae produced by a snail and the total laccase-like activity in the hemolymph of this snail (Pearson’s test, *r* = − 0.33, *P* = 0.017). b Similarly, the hemoglobin rate was negatively correlated to the cercarial output (Pearson’s test, *r* = − 0.54, *P* = 2.528 × 10^−5^). c We also observed a strong positive correlation between the total laccase-like activity and the hemoglobin rate in the hemolymph of the snails. Both of these parameters are good proxies for assessment of snail health (Pearson’s test, *r* = 0.78, *P* = 4.633 × 10^−12^).

## Data Availability

The datasets generated and/or analyzed during the present study are available from the Zenodo repository, 10.5281/zenodo.3466005.

## References

[1] Ewald PW (1993). The evolution of virulence. Sci Am.

[2] Kakehashi M (1996). Populations and infectious diseases: dynamics and evolution. Res Popul Ecol Kyoto.

[3] Lipsitch M, Siller S, Nowak MA (1996). The evolution of virulence in pathogens with vertical and horizontal transmission. Evolution.

[4] Stewart AD, Logsdon JM, Kelley SE (2005). An empirical study of the evolution of virulence under both horizontal and vertical transmission. Evolution.

[5] Alizon S, Hurford A, Mideo N, Van Baalen M (2009). Virulence evolution and the trade-off hypothesis: history, current state of affairs and the future. J Evol Biol.

[6] Jensen KH, Little TJ, Little T, Skorping A, Ebert D (2006). Empirical support for optimal virulence in a castrating parasite. PLoS Biol.

[7] Mennerat A, Hamre L, Ebert D, Nilsen F, Dávidová M, Skorping A (2012). Life history and virulence are linked in the ectoparasitic salmon louse *Lepeophtheirus salmonis*. J Evol Biol.

[8] Schmid-Hempel P (2011). Evolutionary parasitology: the integrative study of infections, immunology, ecology and genetics.

[9] World Health Organization. Schistosomiasis. https://www.who.int/news-room/fact-sheets/detail/schistosomiasis. Accessed 1 Oct 2019.

[10] Maldonado JF, Matienzo JA (1947). The development of *Schistosoma mansoni* in the snail intermediate host, *Australorbis glabratus*. PR J Public Health Trop Med.

[11] Becker W (1980). Metabolic interrelationship of parasitic trematodes and molluscs, especially *Schistosoma mansoni* in *Biomphalaria glabrata*. Z Parasitenkd.

[12] Faro MJ, Perazzini M, Corrêa L dos R, Mello-Silva CC, Pinheiro J, Mota EM (2013). Biological, biochemical and histopathological features related to parasitic castration of *Biomphalaria glabrata* infected by *Schistosoma mansoni*. Exp Parasitol.

[13] Lewis FA, Stirewalt MA, Souza CP, Gazzinelli G (1986). Large-scale laboratory maintenance of *Schistosoma mansoni*, with observations on three schistosome/snail host combinations. J Parasitol.

[14] Gower CM, Webster JP (2004). Fitness of indirectly transmitted pathogens: restraint and constraint. Evolution.

[15] Bayne CJ, Hahn UK, Bender RC (2001). Mechanisms of molluscan host resistance and of parasite strategies for survival. Parasitology.

[16] Moné Y, Ribou A-C, Cosseau C, Duval D, Théron A, Mitta G (2011). An example of molecular co-evolution: reactive oxygen species (ROS) and ROS scavenger levels in *Schistosoma mansoni*/*Biomphalaria glabrata* interactions. Int J Parasitol.

[17] Bonner KM, Bayne CJ, Larson MK, Blouin MS (2012). Effects of Cu/Zn superoxide dismutase (SOD1) genotype and genetic background on growth, reproduction and defense in *Biomphalaria glabrata*. PLoS Negl Trop Dis.

[18] Tavalire HF, Blouin MS, Steinauer ML (2016). Genotypic variation in host response to infection affects parasite reproductive rate. Int J Parasitol.

[19] Gérard C, Moné H, Théron A (1993). *Schistosoma mansoni*–*Biomphalaria glabrata*: dynamics of the sporocyst population in relation to the miracidial dose and the host size. Can J Zool.

[20] Portet A, Pinaud S, Chaparro C, Galinier R, Dheilly NM, Portela J (2019). Sympatric versus allopatric evolutionary contexts shape differential immune response in *Biomphalaria*/*Schistosoma* interaction. PLoS Pathog.

[21] Chevalier FD, Le Clec’h W, Alves de Mattos AC, LoVerde PT, Anderson TJC (2016). Real-time PCR for sexing *Schistosoma mansoni* cercariae. Mol Biochem Parasitol.

[22] Le Clec’h W, Anderson TJC, Chevalier FD (2016). Characterization of hemolymph phenoloxidase activity in two *Biomphalaria* snail species and impact of *Schistosoma mansoni* infection. Parasit Vectors.

[23] Dinguirard N, Cavalcanti MGS, Wu X-J, Bickham-Wright U, Sabat G, Yoshino TP (2018). Proteomic analysis of *Biomphalaria glabrata* hemocytes during *in vitro* encapsulation of *Schistosoma mansoni* sporocysts. Front Immunol.

[24] Lieb B, Dimitrova K, Kang H-S, Braun S, Gebauer W, Martin A (2006). Red blood with blue-blood ancestry: intriguing structure of a snail hemoglobin. Proc Natl Acad Sci USA.

[25] Duvaux-Miret O, Baratte B, Dissous C, Capron A (1991). Molecular cloning and sequencing of the alpha-tubulin gene from *Schistosoma mansoni*. Mol Biochem Parasitol.

[26] Odoemelam E, Raghavan N, Miller A, Bridger JM, Knight M (2009). Revised karyotyping and gene mapping of the *Biomphalaria glabrata* embryonic (Bge) cell line. Int J Parasitol.

[27] Giraldo-Calderón GI, Emrich SJ, MacCallum RM, Maslen G, Dialynas E, Topalis P (2015). VectorBase: an updated bioinformatics resource for invertebrate vectors and other organisms related with human diseases. Nucleic Acids Res.

[28] Marshall OJ (2004). PerlPrimer: cross-platform, graphical primer design for standard, bisulphite and real-time PCR. Bioinformatics.

[29] Jones-Nelson O, Thiele EA, Minchella DJ (2011). Transmission dynamics of two strains of *Schistosoma mansoni* utilizing novel intermediate and definitive hosts. Parasitol Res.

[30] Anderson RM, May RM (1982). Coevolution of hosts and parasites. Parasitology.

[31] Levin BR, Bull JJ (1994). Short-sighted evolution and the virulence of pathogenic microorganisms. Trends Microbiol.

[32] Ebert D (1994). Virulence and local adaptation of a horizontally transmitted parasite. Science.

[33] Ebert D, Herre EA (1996). The evolution of parasitic diseases. Parasitol Today.

[34] Frank SA (1996). Models of parasite virulence. Q Rev Biol.

[35] Coles GC (1971). Haemoglobin changes in infected *Biomphalaria glabrata*. Trans R Soc Trop Med Hyg.

[36] Lee FO, Cheng TC (1972). *Schistosoma mansoni*: alterations in total protein and hemoglobin in the hemolymph of infected *Biomphalaria glabrata*. Exp Parasitol.

[37] Davies CM, Webster JP, Woolhous ME (2001). Trade-offs in the evolution of virulence in an indirectly transmitted macroparasite. Proc Biol Sci.

[38] Cressler CE, McLeod DV, Rozins C, Van Den Hoogen J, Day T (2016). The adaptive evolution of virulence: a review of theoretical predictions and empirical tests. Parasitology.

[39] Boissier J, Morand S, Moné H (1999). A review of performance and pathogenicity of male and female *Schistosoma mansoni* during the life-cycle. Parasitology.

[40] Wang T, Zhao M, Liang D, Bose U, Kaur S, McManus DP (2017). Changes in the neuropeptide content of *Biomphalaria ganglia* nervous system following *Schistosoma* infection. Parasit Vectors.

[41] Le Clec’h W, Dittmer J, Raimond M, Bouchon D, Sicard M (2017). Phenotypic shift in *Wolbachia* virulence towards its native host across serial horizontal passages. Proc Biol Sci.

[42] Dortmans JCFM, Koch G, Rottier PJM, Peeters BPH (2011). Virulence of Newcastle disease virus: what is known so far?. Vet Res.

[43] Kolodny-Hirsch DM, Van Beek NAM (1997). Selection of a morphological variant of *Autographa californica* nuclear polyhedrosis virus with increased virulence following serial passage in *Plutella xylostella*. J Invertebr Pathol.

[44] Wang B, Lee J, Li P, Saberi A, Yang H, Liu C (2018). Stem cell heterogeneity drives the parasitic life cycle of *Schistosoma mansoni*. eLife.

[45] Wang B, Collins JJ, Newmark PA (2013). Functional genomic characterization of neoblast-like stem cells in larval *Schistosoma mansoni*. eLife.

[46] Collins JJ, King RS, Cogswell A, Williams DL, Newmark PA (2011). An atlas for *Schistosoma mansoni* organs and life-cycle stages using cell type-specific markers and confocal microscopy. PLoS Negl Trop Dis.

[47] Zhang P, Sandland GJ, Feng Z, Xu D, Minchella DJ (2007). Evolutionary implications for interactions between multiple strains of host and parasite. J Theor Biol.

[48] Alizon S (2008). Decreased overall virulence in coinfected hosts leads to the persistence of virulent parasites. Am Nat.

[49] Laidemitt MR, Zawadzki ET, Brant SV, Mutuku MW, Mkoji GM, Loker ES (2017). Loads of trematodes: discovering hidden diversity of paramphistomoids in Kenyan ruminants. Parasitology.

[50] Anderson TJC, LoVerde PT, Le Clec’h W, Chevalier FD (2018). Genetic crosses and linkage mapping in schistosome parasites. Trends Parasitol.

