# Supplementary figures and images for: Striking differences in virulence, transmission and sporocyst growth dynamics between two schistosome populations
Source: Parasit Vectors. 2019 Oct 16;12:485. doi: 10.1186/s13071-019-3741-z (PMC6796389; doi:10.1186/s13071-019-3741-z)

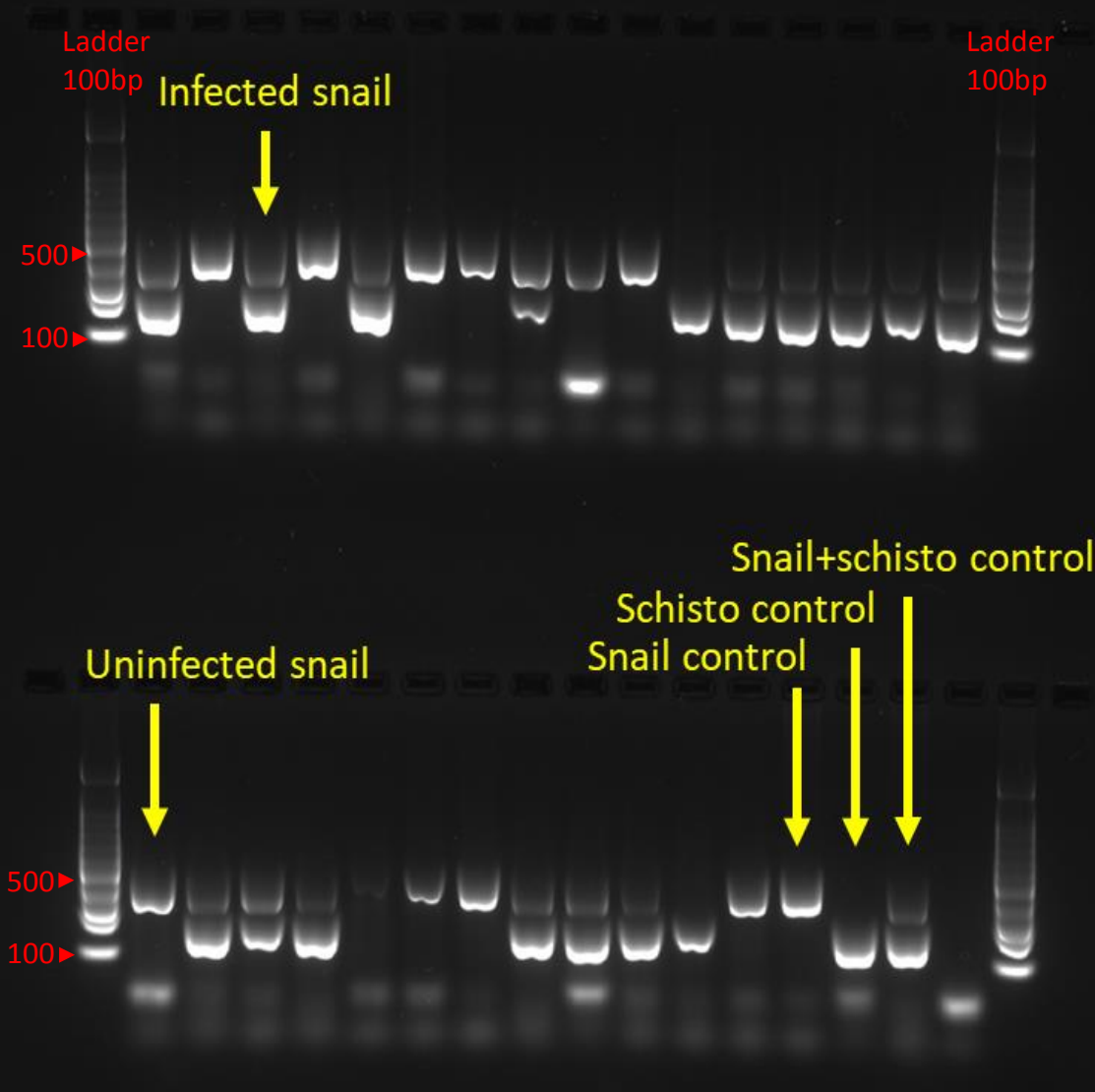

Supplement: Supplementary file 1 — Additional file 1: Figure S1. Multiplex PCR assay for identifying infected prepatent snails. We electrophoresed multiplexed PCR products generated using piwi and α-tubulin-2 primers on 2% agarose gel. The size ladder used is the 100-bp ladder from Promega. Infected B. glabrata Bg26 snails show a “double-band”: a 361-bp piwi snail-specific band and a 190-bp α-tubulin-2 parasite-specific band. Uninfected snails exhibit only the 361 bp piwi snail-specific band, while S. mansoni control show only the 190 bp α-tubulin-2 parasite-specific band. [file 13071_2019_3741_MOESM1_ESM.pdf]

**SmBRE (LS)**

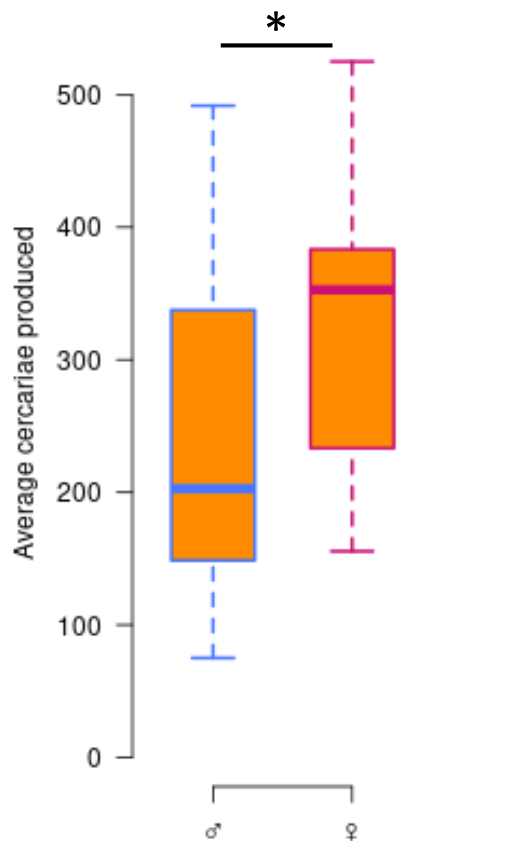

**SmLE (HS)**

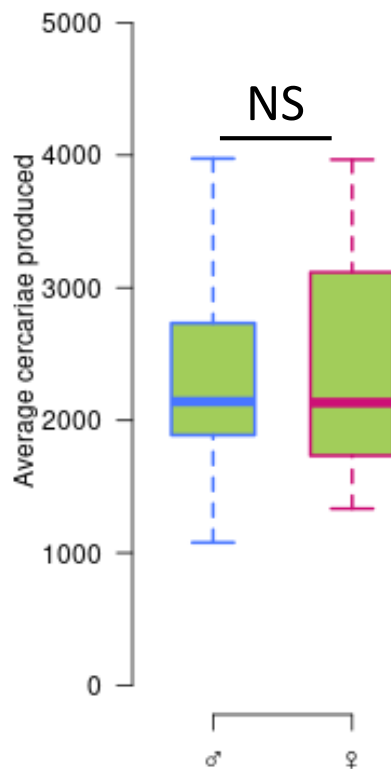

Supplement: Supplementary file 2 — Additional file 2: Figure S2. Impact of S. mansoni sex on the cercarial production. Male sporocysts produced significantly less cercariae than female sporocysts in SmBRE (LS) parasite. There was no difference driven by the sex of the parasites for the SmLE (HS) population of S. mansoni. *P < 0.05, **P ≤ 0.01, ***P ≤ 0.001. [file 13071_2019_3741_MOESM2_ESM.pdf]

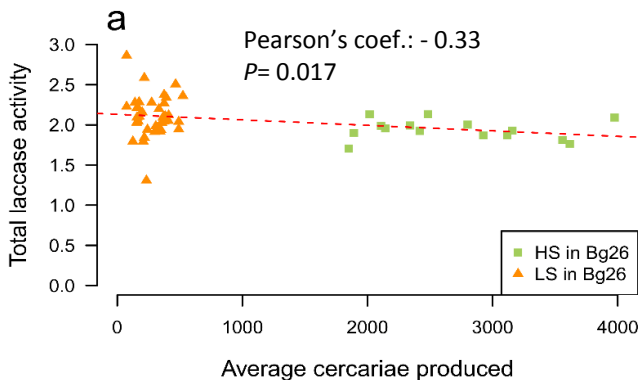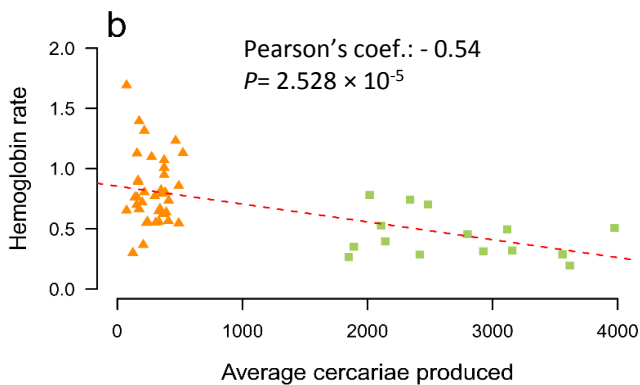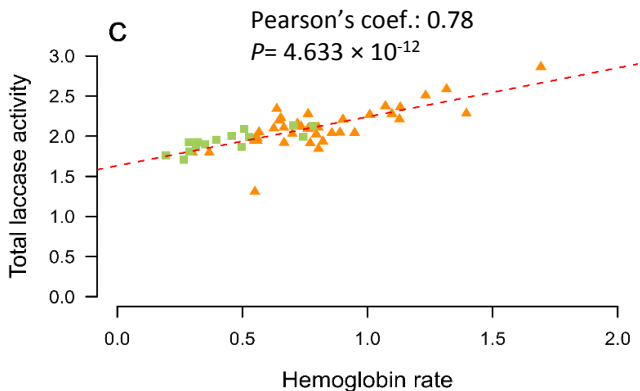

Supplement: Supplementary file 3 — Additional file 3: Figure S3. Virulence of S. mansoni parasites; correlation between cercarial production and measured B. glabrata snail physiological parameters. a There was a negative correlation between the average number of cercariae produced by a snail and the total laccase-like activity in the hemolymph of this snail (Pearson’s test, r = − 0.33, P = 0.017). b Similarly, the hemoglobin rate was negatively correlated to the cercarial output (Pearson’s test, r = − 0.54, P = 2.528 × 10^−5^). c We also observed a strong positive correlation between the total laccase-like activity and the hemoglobin rate in the hemolymph of the snails. Both of these parameters are good proxies for assessment of snail health (Pearson’s test, r = 0.78, P = 4.633 × 10^−12^). [file 13071_2019_3741_MOESM3_ESM.pdf]
